# Steered sample algorithm for acoustic source localization

**DOI:** 10.1371/journal.pone.0241129

**Published:** 2020-10-26

**Authors:** Bin Liu, Lichao Zhang, Pengfei Nie, Xingcheng Han, Yan Han

**Affiliations:** 1 Shanxi Key Laboratory of Information Survey & Processing, North University of China, Taiyuan, China; 2 School of Information and Communication Engineering, North University of China, Taiyuan, China; University of Bradford, UNITED KINGDOM

## Abstract

High-precision source localization depends on many factors, including a suitable location method. Beamforming-based methods, such as the steered response power (SRP), are a common type of acoustic localization methods. However, these methods have low spatial resolution. The SRP method with phase transform (SRP-PHAT) improves the spatial resolution of SRP and is one of the most effective and robust methods for source localization. However, the introduction of a phase transform to SRP might amplify the power of the noise and result in many local extrema in the SRP space, which has a negative impact on source localization. In this paper, a steered sample algorithm (SSA) based on the reciprocity of wave propagation for acoustic source localization is proposed. The SSA localization process is similar to the hyperbolic Radon transform, which is theoretically analyzed and is the most essential difference form the SRP/SRP-PHAT. Compared with the SRP-PHAT, the experimental results demonstrate that the SSA perform better when it comes to array signal positioning with limited array elements and narrow azimuth signal, where SSA can achieve high precision positioning with lower SNR.

## I Introduction

Acoustic localization is widely used in many fields, such as remote sensing, radar, seismic detection, and sonar. Source localization has always been a very active research field, especially for high-precision source localization. At present, the commonly used localization methods can be summarized into two categories: two-step methods based on the time difference of arrival (TDOA) and one-step methods, such as those based on beamforming.

TDOA location methods consist of two steps. The first step is to calculate the TDOA of different sensor pairs, which can be estimated with many methods, such as generalized cross-correlation [1], adaptive delay estimation [2, 3], blind channel identification delay estimation [4], high-order spectral delay estimation [5, 6] and the minimum entropy method [7]. The second step is to estimate the source position by using the estimated delay and prior knowledge. The acoustic source position is determined by the intersections of a series of hyperbolas and is a nonlinear problem. There are two methods for solving this nonlinear problem, namely, maximum likelihood estimation and closed-form estimation. The maximum likelihood estimation is solved using linear approximation and iterative numerical techniques [8]. Most closed-form estimation methods use the least squares principle, which defines an error function through the TDOA and minimizes the error function to determine the source location. Different error functions are defined to achieve source localization with different complexity and performance, such as the spherical cross method [9], spherical interpolation [10], Chan's method [11], etc. The location precision of the two-step method strongly depends on the accuracy of the TDOA estimation, and the positioning error is composed of the TDOA estimation error and the source position estimation error. Any increase of in the two parts of the error can lead to a failure in determining the location. Moreover, the two-step localization method only utilizes the TDOA information and does not make full use of all the information in the obtained signal. The most prominent advantage of the two-step method is effective computation, but the location error might be very large under low signal-to-noise ratio (SNR) conditions [12, 13].

The one-step methods include not only beamforming-based methods, but also subspace algorithms and maximum likelihood estimators [14]. The one-step methods can make full use of all the signal information [15]. In one-step approaches, the most critical issue is the definition or selection of a cost function, which can produce a maximum value at the grid coordinates corresponding to the source position [16]. Many efforts have been devoted to this issue. The most popular one is the steered response power (SRP) method, which is essentially a delay-and-sum beamformer. However, the location performance of the traditional SRP method is poor. Many modified SRP methods have been proposed to improve localization performance by applying filters to array signals. When the phase transform (PHAT) filter is incorporated with the SRP method, the resulting SRP is called the SRP-PHAT [17]. Under noise and reverberation conditions, it is one of the most efficient and robust positioning methods [16, 18]. Moreover, it is suitable for broadband and narrowband array signals and multisource locations [18, 19]. However, the SRP-PHAT has expensive computational cost because the SRP space has many local extrema [17–20]. To reduce the computational cost, many modified methods have been proposed, such as stochastic region contraction [20], coarse-to-fine region contraction [21], stochastic particle filtering [22] and other methods [18, 23–25].

In addition to the aforementioned methods, there are many other new localization approaches, such as time-frequency based method [26], learning based method [27, 28], etc.

For SRP-PHAT, the purpose of introducing PHAT is to broaden the signal spectrum to improve the spatial resolution of SRP. Meanwhile, the PHAT amplifies the noise power and results in many local extrema in SRP the space. In addition, the cross-power/cross-correlation calculations attenuate the energy of sources far away from the array due to the limited sampling time in the SRP-PHAT. To mitigate these issues, a new analogous algorithm, the steered sample algorithm (SSA) based on the reciprocity of wave propagation, is proposed. The basic concept is to regard the sensors as virtual sources. The vibrations generated by the different virtual sources can stack in-phase in the actual source position to form the energy focus. The source can be located by searching for the energy peak. As the SSA makes full use of all the signal information, its localization accuracy and robustness are higher than those of the conventional SRP-PHAT.

The major contributions of our work are as follows:

- A steered sample algorithm based on the reciprocity of wave propagation is proposed for source localization.
- Gaussian kernel is introduced in the SSA to avoid interpolation and reduce the local extremum in the space domain.
- The main difference between the SSA and the better-known SRP-PHAT is proved theoretically; that is, the SSA is similar to the hyperbolic Radon transform, while the SRP-PHAT is not.
- Compared with the SRP-PHAT, the experimental results demonstrate that the SSA can achieve higher localization performance on noisy and reverberation databases and is more robustness under fewer sensors and narrower azimuth acquisition modes.

The remainder of this paper is organized as follows. In Section II, related work is reviewed. In Section III, the proposed steered sample algorithm is introduced in detail. Numerical experiments are carried out on the noisy, reverberation and real speech databases in Sections IV and V. The conclusions are drawn in Section VI.

## II Related work

SRP-PHAT is currently one of the most robust localization methods. In this section, we introduced the SRP-PHAT. The purpose of introducing the SRT-PHAR is to compare it with the proposed algorithm in localization performance.

### Steered response power (SRP)

The SRP method is the output power of the delay-and-sum / filter-and-sum beamformer. SRP localization is used to search for the acoustic source position by steering a sensor array beam to many locations and is based on maximizing the SRP. Hence, SRP localization maximizes the following objective function:

$$W(x)=\sum_{n\in Z}\int_{-\infty }^{+\infty }f_{n}(t+t_{n}(x))dt$$ (1)

where **x** = [*x*,*y*,*z*]^*T*^ denotes a candidate for the acoustic source position; *f*_*n*_(*t*) is the signal acquired by the *n*th sensor, for *n*∈{0,1,…,*N*−1}, where *N* denotes the number of sensors in the array; and *t*_*n*_(**x**) is the time of arrival (TOA) propagation from the source position to the *n*th sensor. In addition, symbol Z denotes the set of integers.

In (1), the item $\sum_{n\in Z}f_{n}(t+t_{n}(x))$ achieves delay-and-sum beamforming, which can be rewritten by the δ-function as follows:

$$\sum_{n\in Z}f_{n}(t+t_{n}(x))=\sum_{n\in Z}f_{n}(t)*\delta (t+t_{n}(x))=\sum_{n\in Z}\int_{-\infty }^{+\infty }f_{n}(\tau )\delta (t+t_{n}(x)-\tau )d\tau .$$ (2)

where the symbol * denotes the linear convolution operator. Substituting (2) into (1), we derive the following:

$$W(x)=\sum_{n\in Z}\int_{t_{n}(x)}^{+\infty }f_{n}(t)dt$$ (3)

It is apparent from (3) that the SRP algorithm is achieved by adjusting the TOA to maximize the objective function *W*(**x**). The integration interval from *t*_*n*_(**x**) to the end time of the signal acquisition is too large, which leads to an oversmoothed SRP and results in low spatial resolution. Especially for multisource situations, the SRP method can only effectively locate the source closest to the sensor array. Other sources far from the sensor array cannot be located due to the oversmoothed SRP. Even for a single-source situation, the TOA calculated from the candidate position near the source is very close to the TOA generated by the actual source, which results in a large energy distribution range in the SRP space. This is also the reason for the low spatial resolution of SRP.

### SRP-PHAT

To improve the spatial resolution of SRP, SRP-PHAT [17] was proposed with narrower SRP peaks. It improves the spatial resolution.

We define

$$Y_{1}(\omega )≜\int_{-\infty }^{+\infty }\sum_{n\in Z}f_{n}(t-t_{n}(x))e^{-i\omega t}dt=\sum_{n\in Z}F_{n}(\omega )e^{-i\omega t_{n}(x)}$$ (4)

where *F*_*n*_(*ω*) is the Fourier transform of signal *f*_*n*_(*t*). According to Pasval’s theorem, the frequency domain form of (1) is as follows:

$$W(x)=\int_{-\infty }^{+\infty }Y_{1}(\omega )\bar{Y_{1}}(\omega )d\omega$$ (5)

where $\bar{Y_{1}}(\omega )$ is the complex conjugate of *Y*_1_(*ω*). If the sampling time is infinite, W (x) is constant and cannot be applied to localization regardless of how the TOA is steered. We thus define

$$Y_{2}(\omega )≜\sum_{m\in Z}F_{m}(\omega )e^{-i\omega t_{m}(x)}$$ (6)

When $\bar{Y_{1}}(\omega )$ is replaced by $\bar{Y_{2}}(\omega )$, Eq (5) can be rewritten as follows:

$$W(x)=\int_{-\infty }^{+\infty }Y_{1}(\omega )\bar{Y_{2}}(\omega )d\omega$$

$$=\sum_{m\in Z}\sum_{n\in Z}\int_{-\infty }^{+\infty }F_{n}(\omega )\bar{F_{m}}(\omega )e^{-i\omega (t_{n}(x)-t_{m}(x))}d\omega$$ (7)

Let *τ*_*n*,*m*_(**x**) = *t*_*n*_(**x**)−*t*_*m*_(**x**), which is the TDOA of a signal emitted at position **x** to sensor *n* and *m*. Then, (7) can be rewritten as follows:

$$W(x)=\sum_{m\in Z}\sum_{n\in Z}\int_{-\infty }^{+\infty }F_{n}(\omega )\bar{F_{m}}(\omega )e^{-i\omega \tau_{n,m}(x)}d\omega$$ (8)

Eq (8) is essentially a cross-SRP. It is well known that the cross-spectrum does not change the frequency content of the signal. Therefore, the spatial resolution of the SRP cannot be improved by (8). To improve the spatial resolution of SRP, a filter should be introduced to broaden the signal spectrum so that the signal becomes a pulse signal. Phase transform [1] is one such filter and is defined as follows:

$$\Psi_{n,m}(\omega )≜\frac{1}{\left|F_{n}(\omega )\bar{F_{m}}(\omega )\right|}$$ (9)

The SRP-PHAT is produced by combining (8) with (9) as follows:

$$W(x)=\sum_{m\in Z}\sum_{n\in Z}\int_{-\infty }^{+\infty }\frac{F_{n}(\omega )\bar{F_{m}}(\omega )}{\left|F_{n}(\omega )\bar{F_{m}}(\omega )\right|}e^{-i\omega \tau_{n,m}(x)}d\omega$$ (10)

The purpose of introducing a phase transform is to broaden the signal spectrum so that the SRP-PHAT can obtain sharper cross-spectrum/cross-correlation peaks and thus improve the spatial resolution of the SRP method. However, the noise is also amplified at the same time, resulting in more local extrema in the SRP space, which makes it difficult to locate the source. The cross-spectral/cross-correlation peak of the sources far from the sensor array is attenuated due to the limited sampling time. The farther away from the array, the more serious the attenuation is, which makes it impossible to locate multiple sources effectively.

## III Proposed method

In this section, we give the signal model of the sound source location from the point of view of the signal and system. On this basis, the localization idea of the proposed algorithm is proposed with the help of the reciprocity of wave propagation. Next, we introduced the basic principles of the proposed algorithm. To avoid errors caused by numerical calculations, we propose an implementation method for the proposed algorithm.

### Signal model

In general, the source localization can be understood as a linear system model, where the input is source signal *s*(*t*) and the output is signal *f*(*t*) collected by the sensor. Therefore, the signals collected by the *n*-th sensor are given by

$$f_{n}(t)=s(t)*h_{n}(t)+\vartheta_{n}(t)$$ (11)

where *h*_*n*_(*t*) is the *n*th channel impulse response and *ϑ*_*n*_(*t*) is the uncorrelated additive background noise. Eq (11) indicates that source signal *s*(*t*) propagates through static medium (linear system) *h*_*n*_(*t*) to produce acquired signal *f*_*n*_(*t*) (system output). According to the reciprocity theorem, the acquired signal *f*_*n*_(*t*), which is the excitation source, propagates to the actual source position in the same propagation mode as the real source to the sensor since the propagation medium is static. If the acquired signals are used as the acoustic source, the in-phase stack occurs at the acoustic source position to form the energy focus. Thus, localization can be achieved by searching for the peak energy.

**Fig 1:**
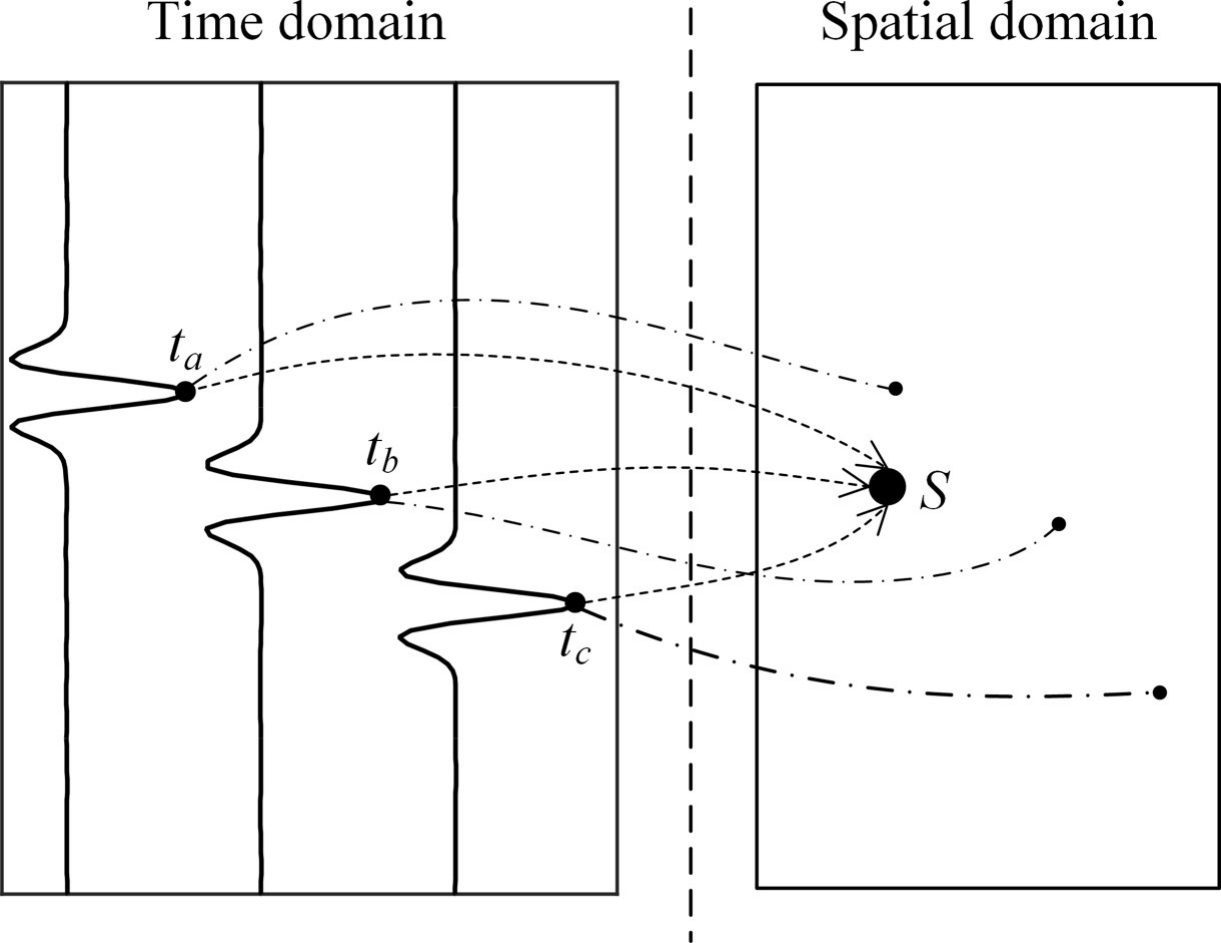
Illustration of the localization concept.

The concept of the SSA is illustrated in Fig 1. When the searched candidate position is the actual source position, the samples of three different collected signals at times *t*_*a*_, *t*_*b*_ and *t*_*c*_ can simultaneously reach source position *S*, and thus, the output energy is the largest. If the searched candidate position is not the source position, the samples at times *t*_*a*_, *t*_*b*_ and *t*_*c*_ are distributed at different positions, and the output energy is relatively small. This process can be seen as transforming the time domain signal into the spatial domain by varying the samples, which is called steered sampling.

### Steered sampling algorithm (SSA)

The well-known time domain sampling process can be described as follows:

$$f(t_{s})=\int_{-\infty }^{+\infty }f(t)\delta (t-t_{s})dt$$ (12)

where *t*_*s*_ is the sampling time and *f*(*t*_*s*_) is the sampled signal of *f*(*t*). Usually, the sampling time of the time domain sampling is equally spaced, that is, *t*_*s*_ = kT, (k = 1,2,…,K), where K is the number of samples and T is the sampling interval. To correlate the time domain sampling with spatial location, let *t*_*s*_ = *t*_*s*_(**x**); then, formula (12) becomes

$$g(x)=f(t_{s}(x))=\int_{-\infty }^{+\infty }f(t)\delta (t-t_{s}(x))dt$$ (13)

The sampling time *t*_*s*_ is changed and is controlled by the spatial position. Therefore, (13) is called steered sampling. We sum the steered samples of all collected signals to form a spatial energy map, as follows:

$$P(x)=\sum_{n\in Z}g_{n}(x)=\sum_{n\in Z}\int_{-\infty }^{+\infty }f_{n}(t)\delta (t-t_{n}(x))dt$$ (14)

where *t*_*n*_(**x**) and g_*n*_(**x**) are steered sampling times and steered samples, respectively. The position corresponding to the maximum of *P*(**x**) is derived from the acoustic source as follows:

$$\tilde{x}=\arg\underset{x\in R^{3}}{\max}P(x)$$ (15)

where $\tilde{x}$ is the estimated source position and the symbol R denotes the set of real numbers.

Compared with (14) and (3), the objective function of the SSA is the superposition of the steered samples, and the objective function of SRP is the superposition of the signals in the rectangular window. The window size is time-varying and determined by *t*_*n*_(**x**). Therefore, the SSA is different from SRP (delay-and-sum beamforming).

A key step in this process is determining *t*_*n*_(**x**) for the source location using the steered samples. One of the simplest and most effective ways is to define *t*_*n*_(**x**) as follows:

$$t_{n}(x)=\frac{\left|x-x_{n}\right|}{v}$$ (16)

where **x**_***n***_ = [*x*_*n*_,*y*_*n*_,*z*_*n*_]^*T*^ is the *n*th sensor position, |**x**−**x**_***n***_| is the distance from the source to the *n*th sensor, and *v* is the velocity.

**Fig 2:**
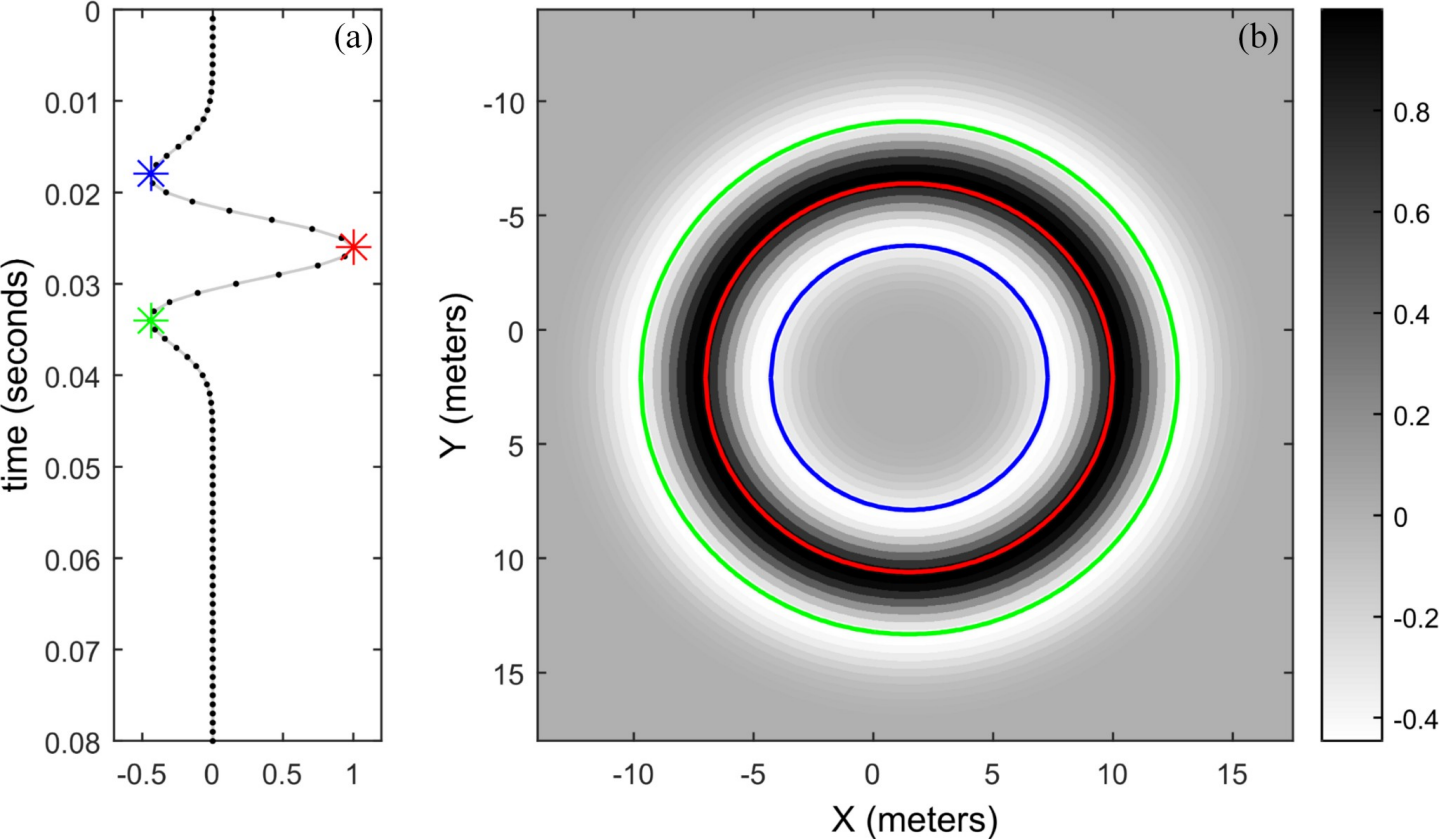
Spatial response of the steered sample. (a) is the acquired signal and (b) is its spatial response.

At a certain sampling time *t*_1_, we can easily see that sample *f*_*n*_(*t*_1_) of the acquired signal is distributed on the spatial circumference with the *n*th sensor position as the center and *t*_1_*v* as the radius. The samples of the entire acquired signal are distributed on a concentric circle with the *n*th sensor position as the center and *tv* as the radius, as shown in Fig 2. Adding the spatial response of all acquired signals together results in a spatial energy map and maximum energy in the real source position. Additionally, the spatial response of the acquired noise also has the same characteristics as the acquired signal, which is also distributed on the concentric circle with a random radius. Therefore, the noise distribution is more dispersed and can be better suppressed when stacking all spatial responses. When the frequency of the collected signals is low, the spatial resolution of the steered sample is reduced, as inferred in Fig 2. The spatial resolution can be improved by whitening the collected signal.

Substitute (16) into (14) to obtain

$$P(x)=\sum_{n\in Z}\int_{-\infty }^{+\infty }f_{n}(t)\delta (t-\frac{\left|x-x_{n}\right|}{v})dt$$ (17)

**Fig 3:**
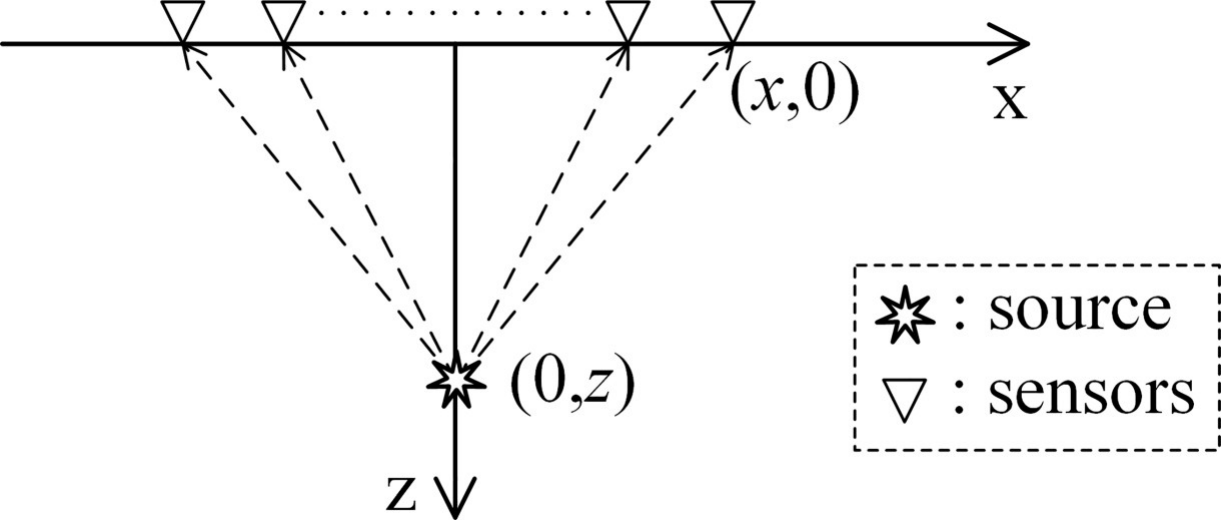
Special geometric relationship between the acoustic source and sensor array.

In the case of a special geometric relationship between the acoustic source and sensor array (see Fig 3), (17) can be expressed as

$$P(x)=\int_{-\infty }^{+\infty }\int_{-\infty }^{+\infty }f(t,x)\delta (t-\frac{\sqrt{x^{2}+z^{2}}}{v})dtdx$$ (18)

where *f*(*t*,*x*) is the signal collected by the sensor at position *x*. Simplify (18) to obtain:

$$P(\tau ,v)=\int_{-\infty }^{+\infty }f(\sqrt{\tau^{2}+\frac{x^{2}}{v^{2}}},x)dx$$ (19)

where τ=zv. This is the hyperbolic Radon transform formula [29]. The only difference is that *v* is variable in the hyperbolic Radon transform, and it is a known constant in SSA. The SSA is similar to the high-dimensional Radon transform for the general geometric relationship between the acoustic source and sensor array. Therefore, the localization idea of the SSA is similar to that of reconstruction. This is the most essential difference between the SSA and the SRP/SRP-PHAT.

### Implementation of the SSA

**Fig 4:**
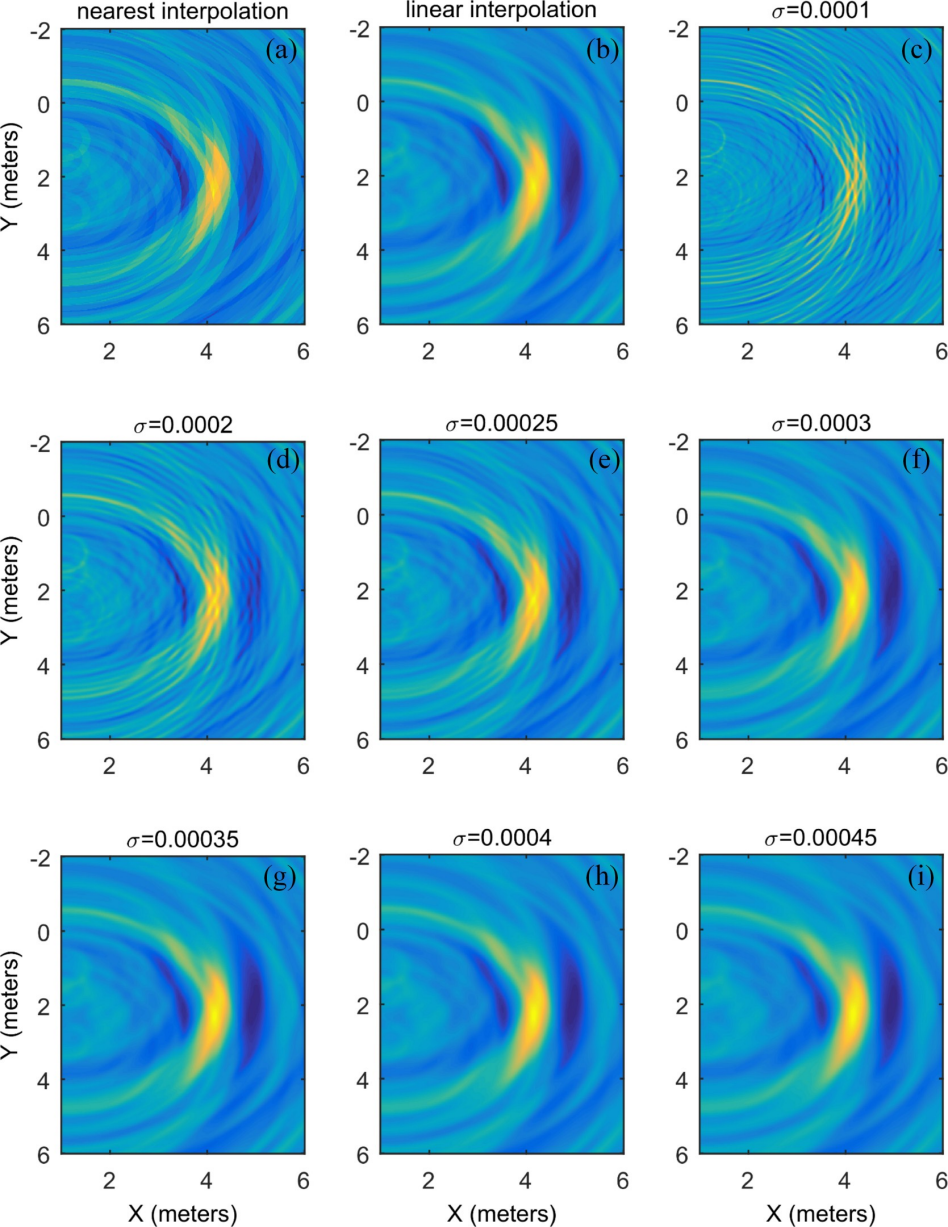
Location energy map using the (a) nearest interpolation, (b) linear interpolation and (c~i) Gaussian function with different widths σ.

Eq (17) can be discretized as follows:

$$P(x)=\sum_{n\in Z}\sum_{m\in Z}f_{n}(t_{m})\delta (t_{m}-\frac{\left|x-x_{n}\right|}{v})$$ (20)

where *t*_*m*_ is the discrete sampling time. Spatial gridding is necessary for the numerical calculation of (20). Assuming that the size of the designated space grid is *n*_*x*_×*m*_*y*_×*l*_*z*_, (20) can be expressed as follows

$$P(k\Delta x)=\sum_{n\in Z}\sum_{m\in Z}f_{n}(t_{m})\delta (t_{m}-t_{n}(k\Delta x))$$ (21)

where ***k*** = (*n*_*x*_,*m*_*y*_,*l*_*z*_), and Δ***x*** = (Δ*x*,Δ*y*,Δ*z*) is the step size of the grid. Steered sampling time *t*_*n*_(***k***Δ***x***) does not always exactly coincide with the time sampled by the sensor. Therefore, interpolation is necessary for computing the steered sample in numerical terms. In this paper, neighborhood interpolation and linear interpolation are analyzed. The steered samples obtained by different interpolation methods are shown in Fig 4A and 4B, which shows that the energy map of linear interpolation is smoother and more continuous than neighborhood interpolation. As a result, the location accuracy is higher, which indicates that linear interpolation can meet the requirements.

Formula (18) only accounts for the current time *t*_*m*_ sample and neglects the relationship among the current time *t*_*m*_, previous time *t*_*m*−1_ and latter time *t*_*m*+1_ samples. Under noisy conditions, this reduces the location accuracy. This problem can be solved by weighting. The purpose of weighting is to achieve better energy focus by not only considering the current moment sample but also the samples of the previous and later moments. A rectangular window is a natural choice, but a rectangular window can make the energy map smooth, causing an energy focus blur and reducing the location accuracy. The ideal weighting function places the largest weight value at the current moment and decreases the weight values at the previous and later moments. The Gaussian function is such a weighted function and satisfies these requirements.

Gaussian functions are widely used in mathematics, engineering and other fields [30]. A Gaussian function is formed as follows:

$$g(x)=ae^{-\frac{{\left(x-b\right)}^{2}}{2\sigma^{2}}}$$ (22)

where *a*,*b*,*σ* are arbitrary real constants. Parameter *a* is the height of the Gaussian function peak, *b* is the center of the peak and *σ* controls the width of the Gaussian function. When parameter *a* equals 1 and *σ* tends to 0, the Gaussian function tends to the δ-function, as follows

$$\underset{\sigma \to 0}{\lim}e^{-\frac{{\left(x-b\right)}^{2}}{2\sigma^{2}}}=\delta (x-b)$$ (23)

According to (20), formula (18) can be rewritten as follows

$$P(k)=\underset{\sigma \to 0}{\lim}\sum_{n\in Z}\int_{-\infty }^{+\infty }f_{n}(t)e^{-\frac{{\left(t-t_{n}(k\Delta x)\right)}^{2}}{2\sigma^{2}}}dt$$ (24)

When *σ* tends to zero, (21) can exactly match (18). The integral term can be seen as a convolution of *f*_*n*_(*t*) and the Gaussian function in (16). Thus, it has a low-pass filtering function that suppresses high-frequency random noise.

The introduction of the Gaussian function avoids interpolation in the steered sample process. The location energy map of the noisy modeling data under the different width parameter *σ* condition is shown in Fig 4C–4I. It shows that when width *σ* is smal (*σ* = 0.0001), the energy is scattered, and it is difficult to determine the maximum, resulting in inaccurate localization. With an increase in width *σ*, the energy is gradually concentrated, and the energy is the most concentrated when *σ* = 0.00025. The noise interference decreases, and the energy map is smoother as width *σ* increases further. The difference in the energy is not obvious around the energy peak. It is difficult to accurately determine the position of the energy peak, which leads to reduced localization accuracy. When width *σ* = 0.003, the energy map is almost the same as that of linear interpolation.

## IV Numerical experiments

**Fig 5:**
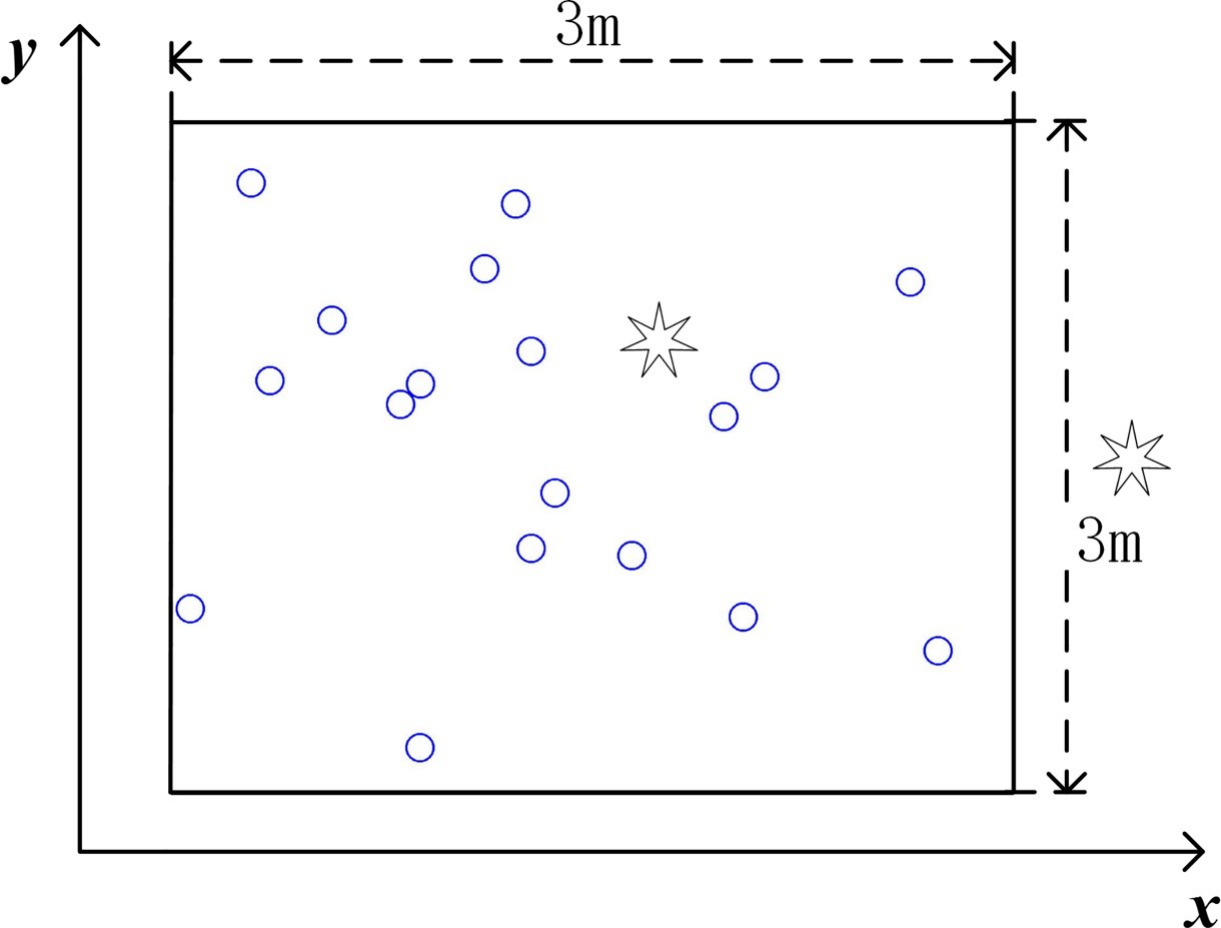
The layout of the sensors and source. “○” represents the sensors, “
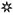
” represents the acoustic source.

Because of the nonrepeatability and nonverifiability of real events in some cases (such as an opaque medium or complex environment), it is difficult to measure the error between the calculated and real source positions. Thus, synthetic data are employed to verify the performance of the SSA. The synthetic tests, with controllable errors in the input data, are flexible when comparing the performance of localization methods under different conditions. Meanwhile, a 2D situation is analyzed to conveniently represent the positioning results and the correlation analysis, that is, the sensors and the source are located on the same plane. Without loss of generality, the sensors are randomly laid in a 3×3 m range plain. The sensors and source locations are shown in Fig 5. The localization performance of single-source and multisource scenarios are analyzed. For the single source scenarios, in which the acoustic sources are located inside and outside the sensor range are discussed. The source localization results are compared with those of the SRP-PHAT [17] for full space scanning.

### Synthetic data

The synthetic data are obtained through the signal model established by (11). In the signal model, the signal generated by the acoustic source is a broadband signal, as follows:

$$s(t)=[1-2a^{2}t^{2}]e^{-a^{2}t^{2}}$$ (25)

where a = *πf*_0_ and *f*_0_ is the dominant frequency. The *f*_0_ = 200 Hz when generating synthetic data. The impulse response function is as follows:

$$h_{n}(t)=\delta \left(t-\frac{\left|x-x_{n}\right|}{v}\right)$$ (26)

where *v* = 340 m/s. Therefore, the synthetic data for the single source are as follows:

$$f_{n}(t)=s(t)*h_{n}(t)=[1-2a^{2}t^{2}]e^{-a^{2}t^{2}}*\delta \left(t-\frac{\left|x-x_{n}\right|}{v}\right)$$ (27)

The synthetic data of multiple sources are as follows:

$$f_{n}(t)=[1-2a^{2}t^{2}]e^{-a^{2}t^{2}}*\sum_{i=1}^{N}\delta (t-\frac{\left|x_{i}-x_{n}\right|}{v})$$ (28)

where N is the number of acoustic sources and **x**_***i***_ is the position of the *i-*th acoustic source.

### Single source experiment

Five sensors are randomly laid in a range of 3×3 m. The source is located within the sensor range, and its coordinates are (1.5 m, 1.1 m). White Gaussian noise is added to the synthetic data to make the signal-to-noise ratio (SNR) equal to -5 dB.

Two cases are analyzed in this subsection. In the first case, the acoustic source is inside the range of the sensor distribution, which is used to locate the narrow azimuth signals. In the second case, the acoustic source is outside the range of sensor distribution, which is used to locate the wide azimuth signals. The sensors can receive a full azimuth signal from the acoustic source in the first case. Only limited azimuth signals can be received in the second case. The purpose of studying these cases is to analyze the robustness of the SSA and SRP-PHAT to limited azimuth signals.

**Fig 6:**
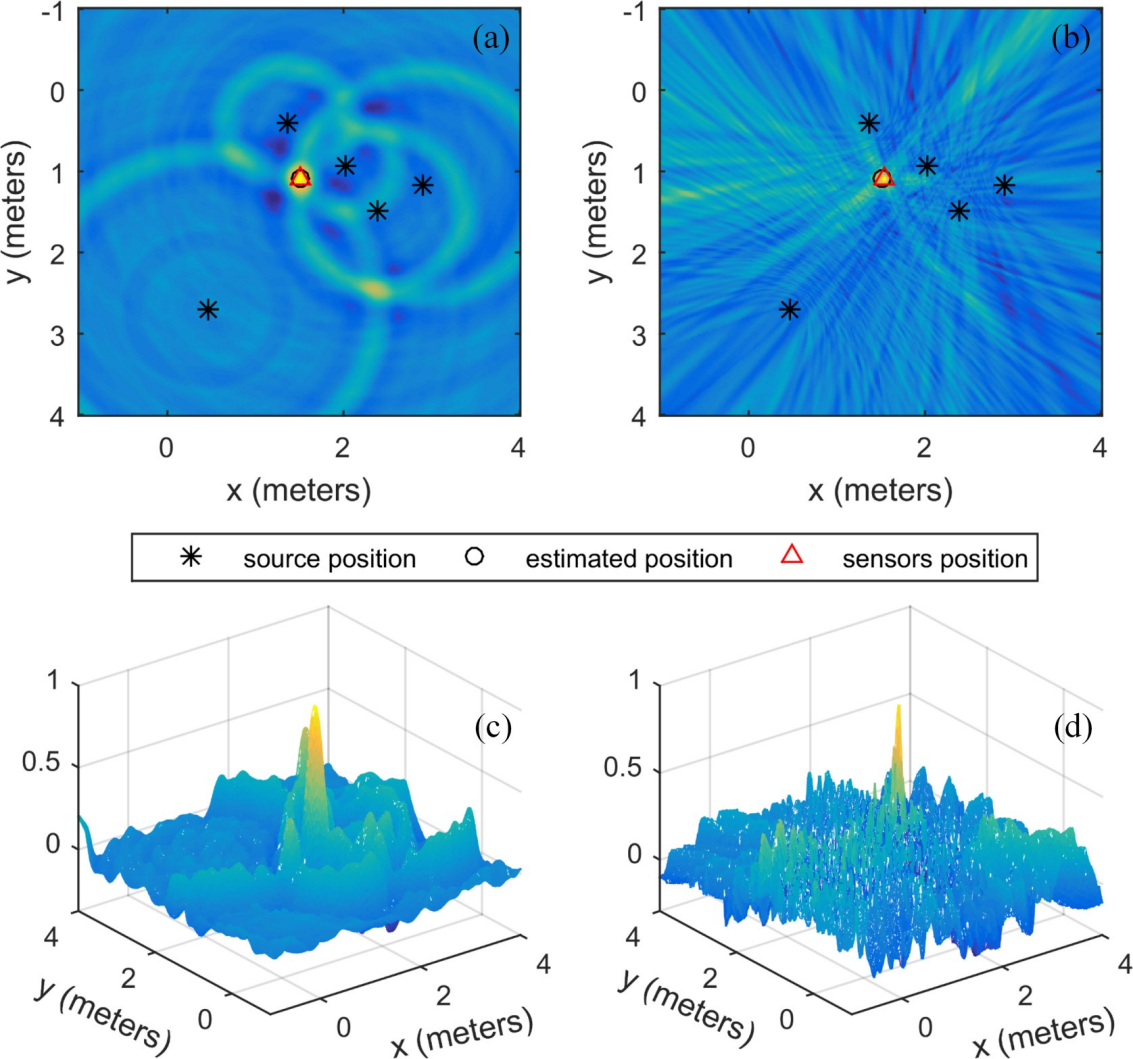
The energy map of the source inside the sensor distribution range. (a) the SSA, (b) the SRP-PHAT, and (c) and (d) are the 3D displays of (a) and (b), respectively.

For the first case, the energy maps of the SSA and the SRP-PHAT are shown in Fig 6A–6D. Fig 6A and 6C and Fig 6B and 6D are the 2D and 3D energy maps for the SSA and the SRP-PHAT, respectively. The positioning result is (1.5 m, 1.1 m) for the SSA and (1.5 m, 1.08 m) for the SRP-PHAT. The positioning result of the SSA is exactly the same as the given source coordinate. The positioning result of the SRP-PHAT has only a small error in the y-coordinate, and the overall error is very small. Fig 6C and 6D shows that the energy map of the SSA is relatively smooth and the local extremum is less than that of the SRP-PHAT. Although the energy peak of the SRP-PHAT is sharper than that of the SSA and the energy is more concentrated, the apparent sharp peak is not the true source position. In general, the positioning results of the two methods both have high accuracy. Therefore, the SSA and SRP-PHAT have similar positioning performances for wide azimuth array signals, but the signal-to-noise ratio of the spatial spectrum of the SSA is higher.

**Fig 7:**
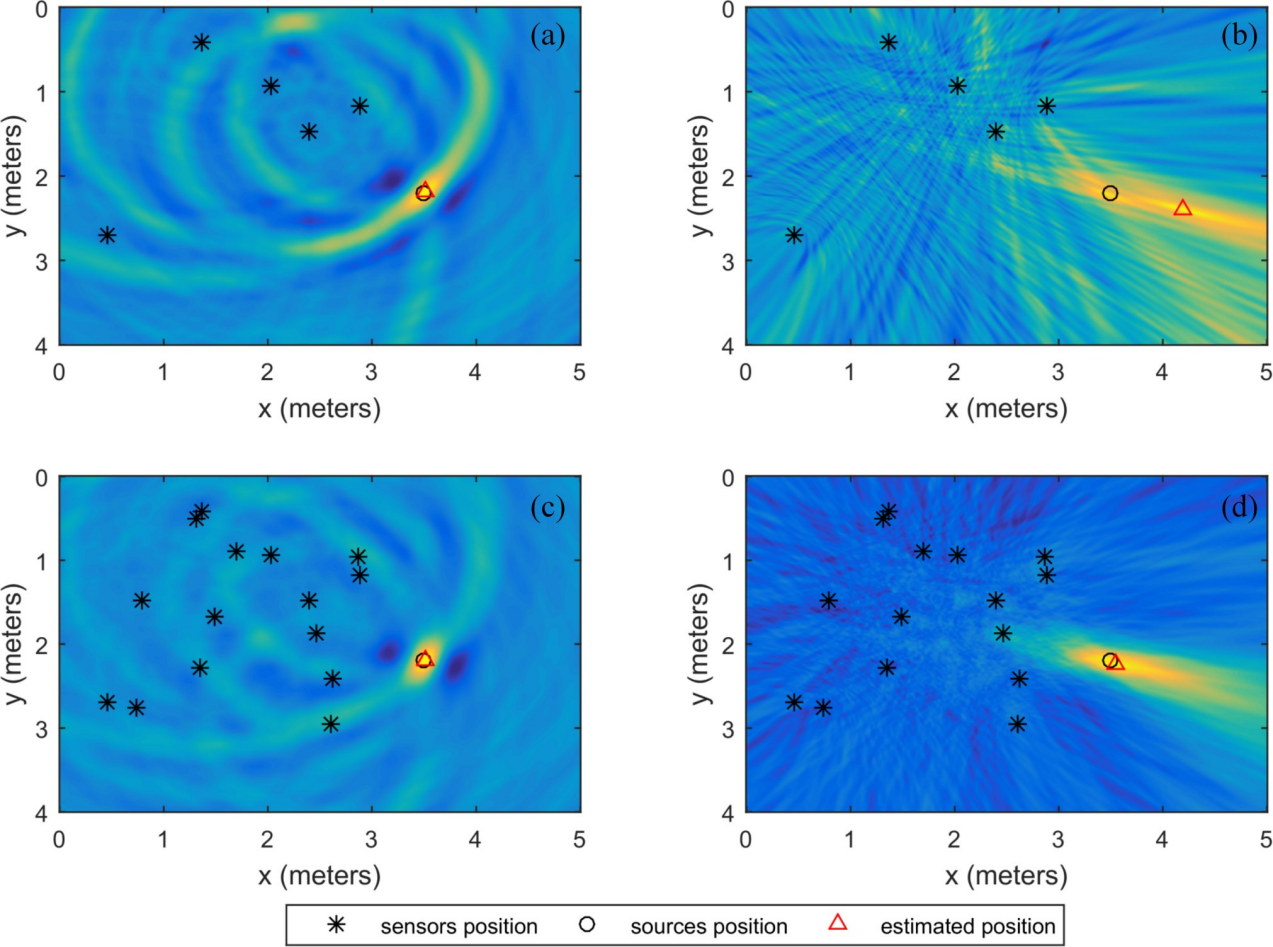
The location energy map for the source located outside the sensor distribution area: (a) and (b) are the energy maps of the SSA and the SRP-PHAT for 5 sensors, respectively; (c) and (d) are the energy maps of the SSA and the SRP-PHAT for 15 sensors, respectively.

For the second case, the coordinates of the source are (3.5 m, 2.2 m). The other simulation parameters are the same as in the previous experiment. The energy maps of the SSA and the SRP-PHAT are shown in Fig 7A and 7B, respectively. The localization result of the SSA is (3.52 m, 2.18 m) and that of the SRP-PHAT is (4.2 m, 2.4 m). The localization results of the two methods are marked in Fig 7 with a red triangle. The localization results show that the localization accuracy of the SSA is much higher than that of the SRP-PHAT. Combined with the analysis in Fig 6, the SSA is more suitable for narrow azimuth array signals than the SRP-PHAT. To improve the positioning results of the SRP-PHAT for narrow azimuth array signals, it is necessary to increase the number of sensors. Fig 7C and 7D shows the energy map of the SSA and the SRP-PHAT when the number of sensors is increased from 5 to 15. The corresponding localization result is (3.51 m, 2.18 m) for the SSA and (3.56 m, 2.24 m) for the SRP-PHAT. It can be seen from Fig 7C and 7D that the energy aggregation of the SRP-PHAT is enhanced significantly with the increase in the number of sensors, and the location result is improved significantly (see Table 1). The energy aggregation of the SSA has been improved, but not as obviously as that of the SRP-PHAT, and the localization result is almost unchanged (see Table 1). The SSA is not sensitive to the number of sensors, while the SRP-PHAT is sensitive to the number of sensors. This result shows that the SSA is more suitable for narrow azimuth array signals than the SRP-PHAT.

**Table 1:** The localization result of the SSA and the SRP-PHAT for two cases.

	Inside		Outside			
			5 sensors		15 sensors	
	x(m)	y(m)	x(m)	y(m)	x(m)	y(m)
**Preset coordinate**	1.5	1.1	3.5	2.2	3.5	2.2
**SRP-PHAT**	1.5	1.08	4.2	2.4	3.56	2.24
**SSA**	1.5	1.1	3.52	2.18	3.51	2.18

Compared with the SRP-PHAT, the SSA can achieve high-precision positioning under the limited azimuth information and fewer sensors. However, the positioning performance of the two methods is similar when it comes to wide azimuth array signal.

### Multisource experiment

**Fig 8:**
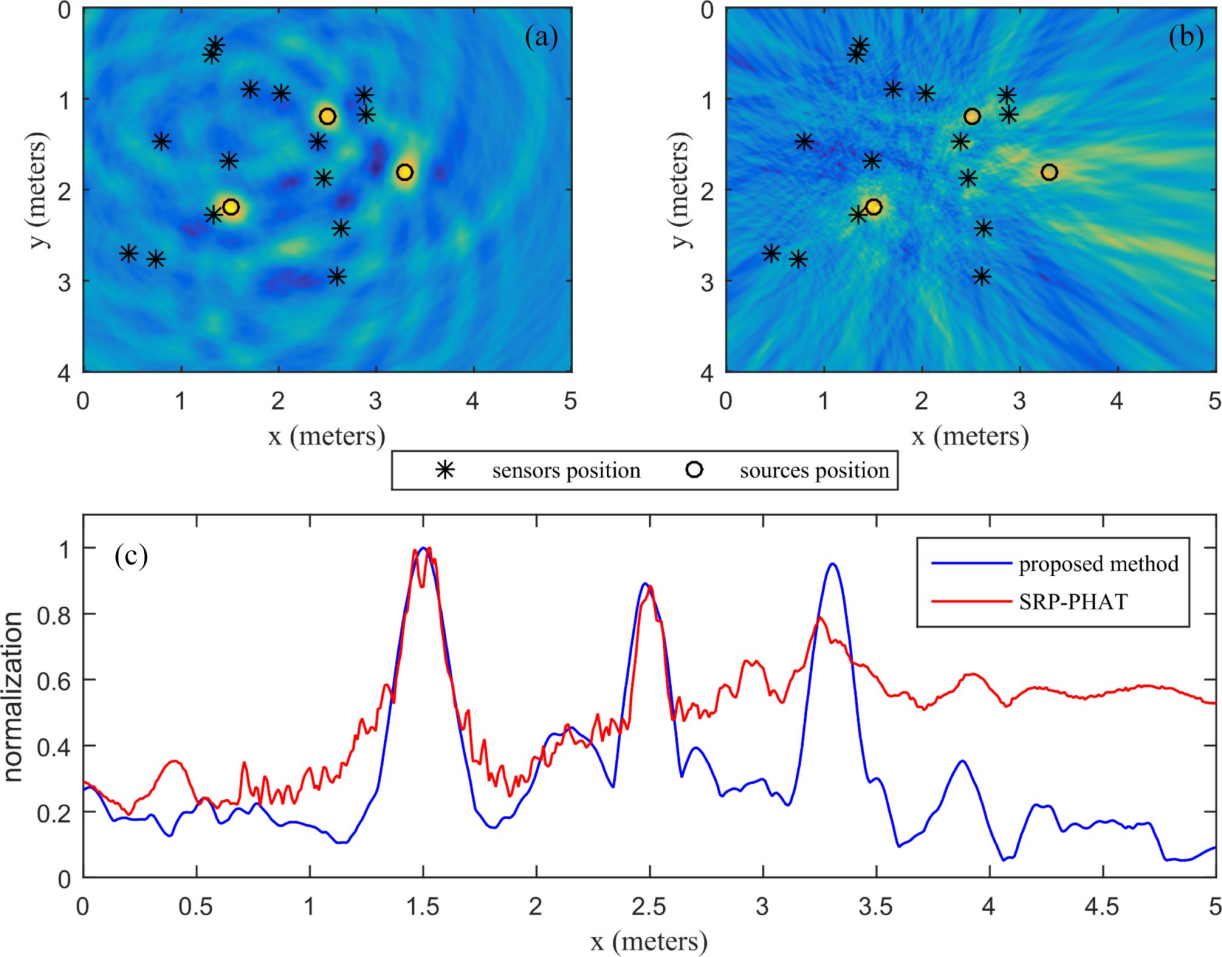
The energy maps for 3 sources: (a) the SSA, (b) the SRP-PHAT and (c) the y-direction normalized average energy.

For the multisource case, the sensor layout method and other parameters are the same as those of the single source, except for the number of sources. There are three sources in space, two of which are distributed within the sensor range and the other outside the sensor range. The coordinates are (1.5 m, 2.2 m), (2.5 m, 1.2 m) and (3.3 m, 1.8 m). In the case of the three sources, the localization energy maps of the SSA and the SRP-PHAT using 15 sensors are shown in Fig 8A and 8B. As shown in Fig 8A, the positioning energy of the SRP-PHAT is more dispersed and has many extrema. Therefore, it is difficult for SRP-PHAT to locate the source. However, the energy concenration of the SSA is better, and the energy accumulation region is essentially coincident with the position of the source in Fig 8B. Fig 8C is the normalized average energy in the y-direction, from which it can be seen that the positioning energy of the SSA is almost the same for the three sources. The positioning energy of the SRP-PHAT is weakened for the source (3.3 m, 1.8 m) away from the sensors. Moreover, this energy is close to the surrounding energy, which may lead to the localization effect of this source (3.3 m, 1.8 m). The positioning errors of the three sources are 0.01, 0.0183, and 0.01 m for the SSA and 0.05, 0.01, and 0.0539 m for the SRP-PHAR, respectively. Therefore, in the multisource case, the SSA is superior to the SRP-PHAT.

### Error analysis

Error analysis is performed through Monte Carlo simulations. The location errors under different SNRs and different sensor numbers are discussed. The location error is defined as the distance from the actual source to the estimated source.

**Fig 9:**
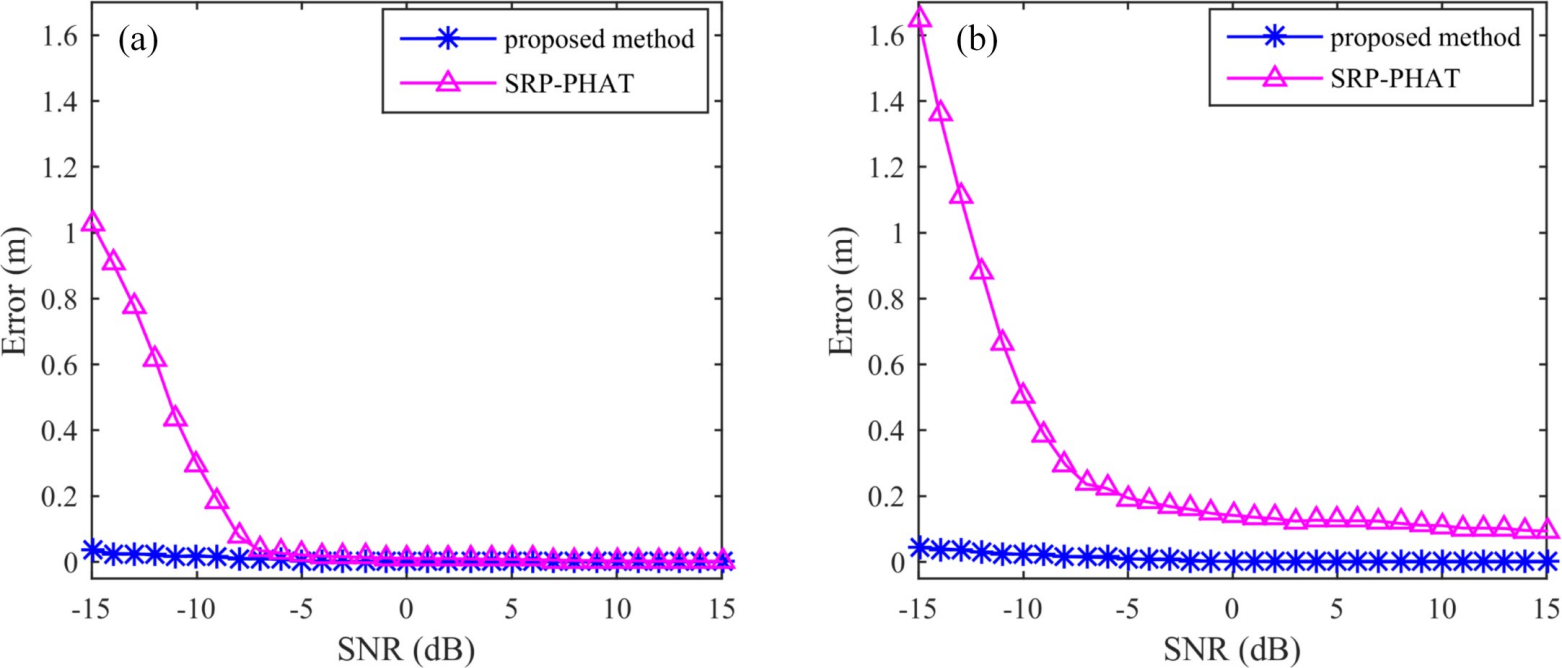
Location error under different SNRs: (a) the source position is inside the sensor range and (b) the source position is outside the sensor range.

Under the different SNRs and the same number of sensor (5 sensors) conditions, the localization errors of the SSA and the SRP-PHAT are shown in Fig 9A and 9B for the two cases where the source is inside and outside the sensor distribution range. Fig 9A and 9B indicates that the localization errors of the SSA are small both inside and outside the sensor distribution range, and the error changes slowly when the SNR changes from -15 dB to 15 dB. However, the localization error of the SRP-PHAT varies greatly when the SNR changes from -15 dB to 15 dB. In comparison, when the source is located outside the sensor distribution range, the localization error changes more dramatically with the change in SNR. Under the same conditions, the localization error of the SRP-PHAT for the source inside the sensor distribution range is less than the source outside the sensor distribution range. When the SNR is less than -10 dB, the location error of the SRP-PHAT is large, while the location error of the SSA is obviously smaller than that of the SRP-PHAT, whether the source is located inside or outside the sensor distribution range. It is shown that the SSA can be applied to high-precision positioning with lower SNRs compared to the SRP-PHAT.

**Fig 10:**
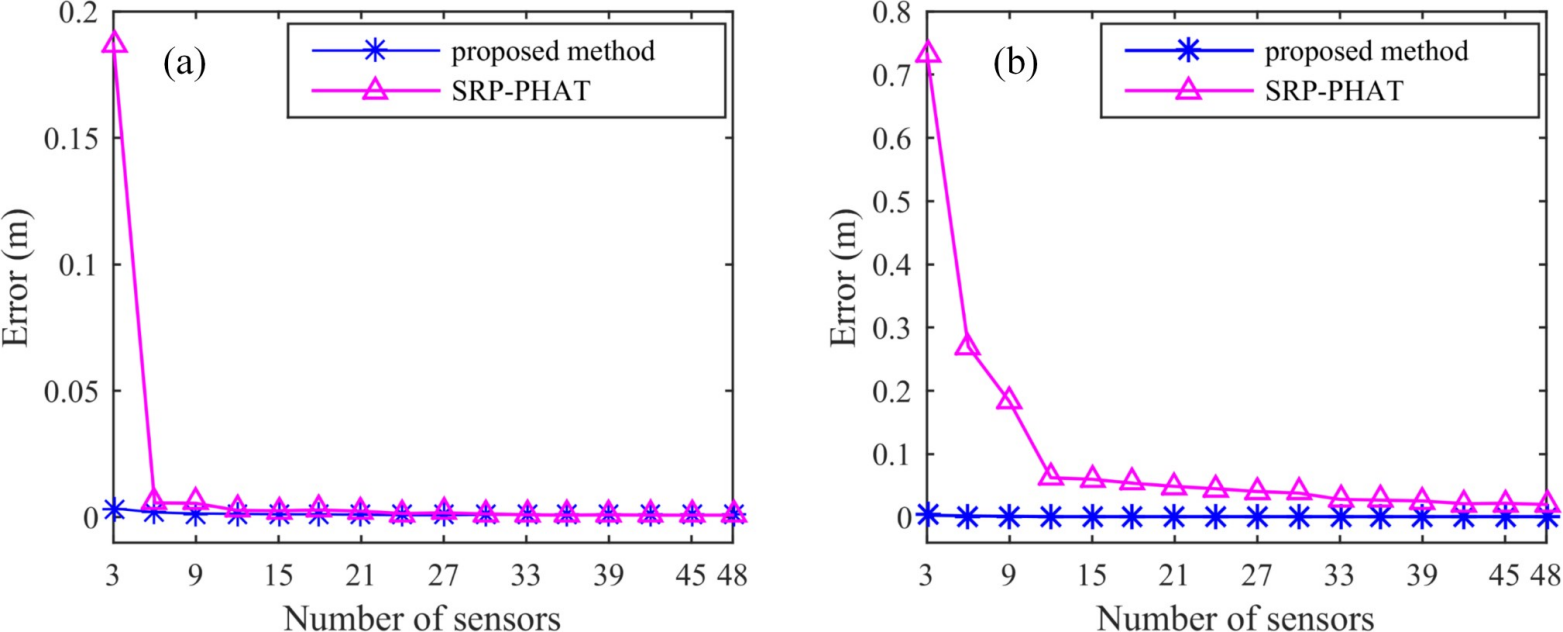
Location error under different numbers of sensors, (a) the source position is inside the sensor distribution range and (b) the source position is outside the sensor distribution range.

Under different numbers of sensors and the same SNR conditions, the localization errors of the SSA and the SRP-PHAT are shown in Fig 10A and 10B in the two cases where the source is inside and outside the sensor distribution range. When the source is located inside the sensor distribution range, the localization error changes as the number of sensors increases from 3 to 48, as shown in Fig 10A. It shows that the localization error of the SSA and the SRP-PHAT is small and not more than 10 cm. The difference is that the SRP-PHAT has a large localization error when there are few sensors, and the localization error gradually decreases as the number of sensors increases; however, the localization error of the SSA remains very small with an increase in the number of sensors. When the acoustic source is outside the sensor distribution range, the increase in the number of sensors greatly improves the localization error of the SRP-PHAT, but the error variation of the SSA is still very small, as shown in Fig 10B. The error analysis shows that the SSA can achieve highly precise localization in the presence of a few sensors, whether or not the source is inside the sensor distribution range or not.

A defined error is noted when the distance from the estimated source to the actual source is an absolute amount. It does not take into account the relativity between the defined error and the distance from the actual source to the reference point, which causes the localization error to appear to be small, even if the localization accuracy is not high. For example, assuming that the localization error is 0.01 m and the distance from the actual source to the reference point is 0.05 m, the localization error is small under this assumption, but the actual localization accuracy is not high. Therefore, we introduce the error rate as defined below:

$$Er=\frac{\left|x-\tilde{x}\right|}{\left|x\right|}\times 100\%$$ (29)

The average error and the error rate with different signal-to-noise ratios of the experimental results for different SNRs and different numbers of sensors are shown in Table 2. The average error and the error rate of the SSA are obviously smaller than those of the SRP-PHAT, regardless of the change in the SNR or the number of sensors. This indicates that the SSA is not sensitive to white noise and provides strong robustness. Furthermore, the SSA can achieve high-precision location with fewer sensors.

**Table 2:** Average error and error rate.

		SNR variation		Sensor quantity variation	
		AveEr (m)	Er (%)	AveEr (m)	Er (%)
**Inside**	**SSA**	0.009	0.48	0.001	0.054
	**SRP-PHAT**	0.258	13.87	0.013	0.7
**Outside**	**SSA**	0.010	0.24	0.005	0.12
	**SRP-PHAT**	0.489	11.98	0.119	2.92

AveEr represents the average error. Er represents the error rate.

## V Location performance in reverberation and real environments

In the previous section, the positioning performance of the SSA for the simulation data constructed by the time-distance relationship is analyzed and did not consider the reverberation effect. However, reverberation is one of the main factors affecting positioning in many cases. In this section, the positioning performance of two acoustic sources in a reverberant environment is analyzed. Finally, the effectiveness of the SSA is verified by an actual speech signal.

The image source technique [31] is a common method for evaluating the performance of acoustic source localization in reverberant environments. The mirror source technique is employed to simulate the room impulse response. The room dimension is R = [3.2 m, 4 m, 2.7 m]^T^, and the reflection coefficients for each surface are 0.73, 0.55, 0.78, 0.73, 0.48, and 0.62. The reverberation time is RT60 = 0.6 s, and the sampling frequency is 16 kHz.

### Performance in reverberation environments

**Fig 11:**
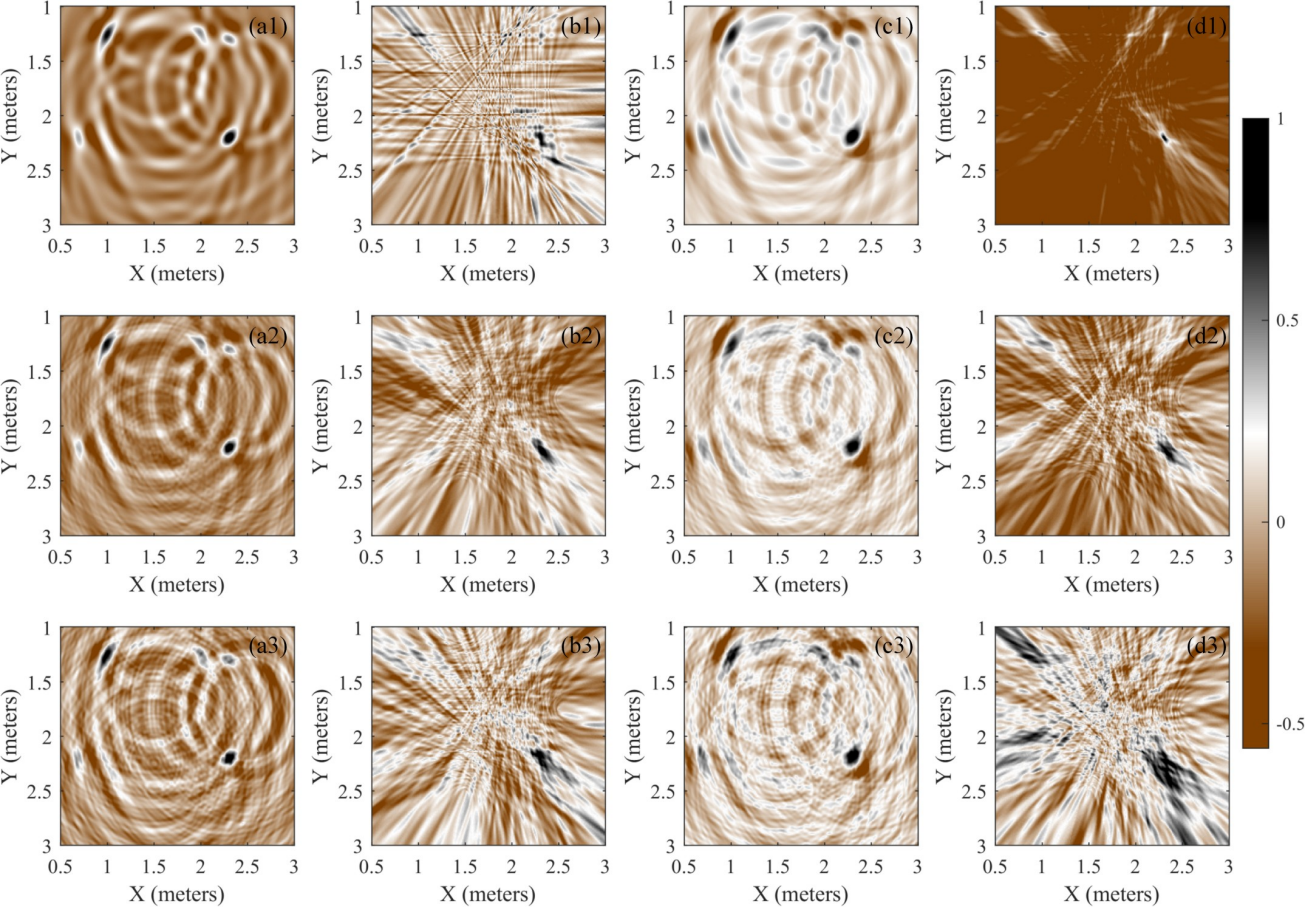
2D positioning results of the SSA and SRP-PHAT under reverberation conditions. a1~d1 are the localization results of noise-free data, a2~d2 are the localization results of noisy data with a SNR of -2 dB, and a3~d3 are for a SNR of -5 dB. a1~a3 are the localization results of the SSA for the narrowband acoustic source, and b1~b3 are for the SRP-PHAT; c1~c3 are the location results of the SSA for the wideband acoustic source, and d1~d3 are for the SRP-PHAT.

The 2D positioning in a reverberant environment is first analyzed; that is, both the acoustic source and receiver are in the same plane of z = 1 m. The receivers are arranged in a 5-element cross array, and the coordinates of the acoustic sources are p_s1_ = [2.3 m, 2.2 m, 1 m]^T^ and p_s2_ = [1 m, 1.25 m, 1 m]^T^. The acoustic source signals are a wideband signal and a narrowband sinusoidal signal, respectively, as shown in Eq (25). The spatial scanning resolution of the SSA and SRP-PHAT is 0.01 m in all directions of X, Y and Z. By adding white Gaussian noise to the composite signal, noisy signals with signal-to-noise ratios (SNRs) of -2 dB and -5 dB can be obtained. Then, the noise-free data and noisy data with SNRs of -2 dB and -5 dB are used for positioning with the SSA and SRP-PHAT. The energy maps are shown in Fig 11. The positioning results (source coordinates) are listed in Table 3, and the unit used is meters. The positioning results verify the conclusion of the above analysis. The average error (AveEr) illustrates that the SSA is suitable for both wideband and narrowband signals, and the SRP-PHAT is more suitable for wideband signals.

**Table 3:** Location 2D results of the two sound sources in reverberation.

Source type	algorithm	Dataset			AveEr
		Noise free	-2 dB	-5 dB	
**narrowband**	**SSA**	1.0, 1.25	1.0, 1.24	0.99, 1.25	0.007
		2.3, 2.2	2.3, 2.2	2.32, 2.19	0.02
	**SRP-PHAT**	2.45, 2.41	2.28, 2.35	2.22, 2.18	1.59
		2.3, 2.19	2.31, 2.21	2.29, 2.25	0.03
**wideband**	**SSA**	1.0, 1.25	1.02, 1.24	1.01, 1.25	0.01
		2.3, 2.2	2.29, 2.2	2.3, 2.16	0.02
	**SRP-PHAT**	1.0, 1.25	1.01, 1.25	1.69, 1.72	0.28
		2.3, 2.2	2.34, 2.24	2.35, 2.17	0.04

From the analysis of Fig 11 and Table 3, the following knowledge can be concluded. 1). Under the reverberation condition, the SRP-PHAT has a better positioning performance for wideband acoustic sources than for narrowband acoustic sources, and the energy concentration at the acoustic source position is high under high SNR conditions, as shown in Fig 11(d1). 2). The SRP-PHAT has poor positioning performance of multiple narrowband acoustic sources because of the existence of many local extreme values, as shown in Fig 11(b1). 3). Under the reverberation condition, the SRP-PHAT does not perform well in the positioning of low SNR data, where it almost loses the ability of acoustic source positioning at the location of (1 m, 1.25 m), as shown in Fig 11(b2), 11(d2), 11(b3) and 11(d3). This might be due to the weak energy and the large distance between the acoustic source and sensor array. Moreover, the existence of many local extreme values in the SRP space causes serious interference with acoustic source energy and the loss of positioning ability. 4). Under the reverberation condition, the SSA has better positioning performance for both wideband acoustic sources and narrowband acoustic sources. Relatively speaking, the SSA performs better for wideband acoustic sources, and it can achieve good positioning for two acoustic sources, as shown in Fig 11(a1) and 11(c1). 5). Under the reverberation condition, the anti-noise ability of the SSA is better, and it achieves a good positioning result when the SNR is -5 dB, as shown in Fig 11(a3) and 11(c3). 6). Local extreme values similar to those in the SRP-PHAT also exist in the SSA, but the low number and weak energy of these local extreme values lead to a smaller influence on positioning, as shown in Fig 11(a1)~11(a3). 7). The energy aggregation at the acoustic source of the SSA is not as good as that of the SRP-PHAT, which can be improved by whitening the collected data.

**Fig 12:**
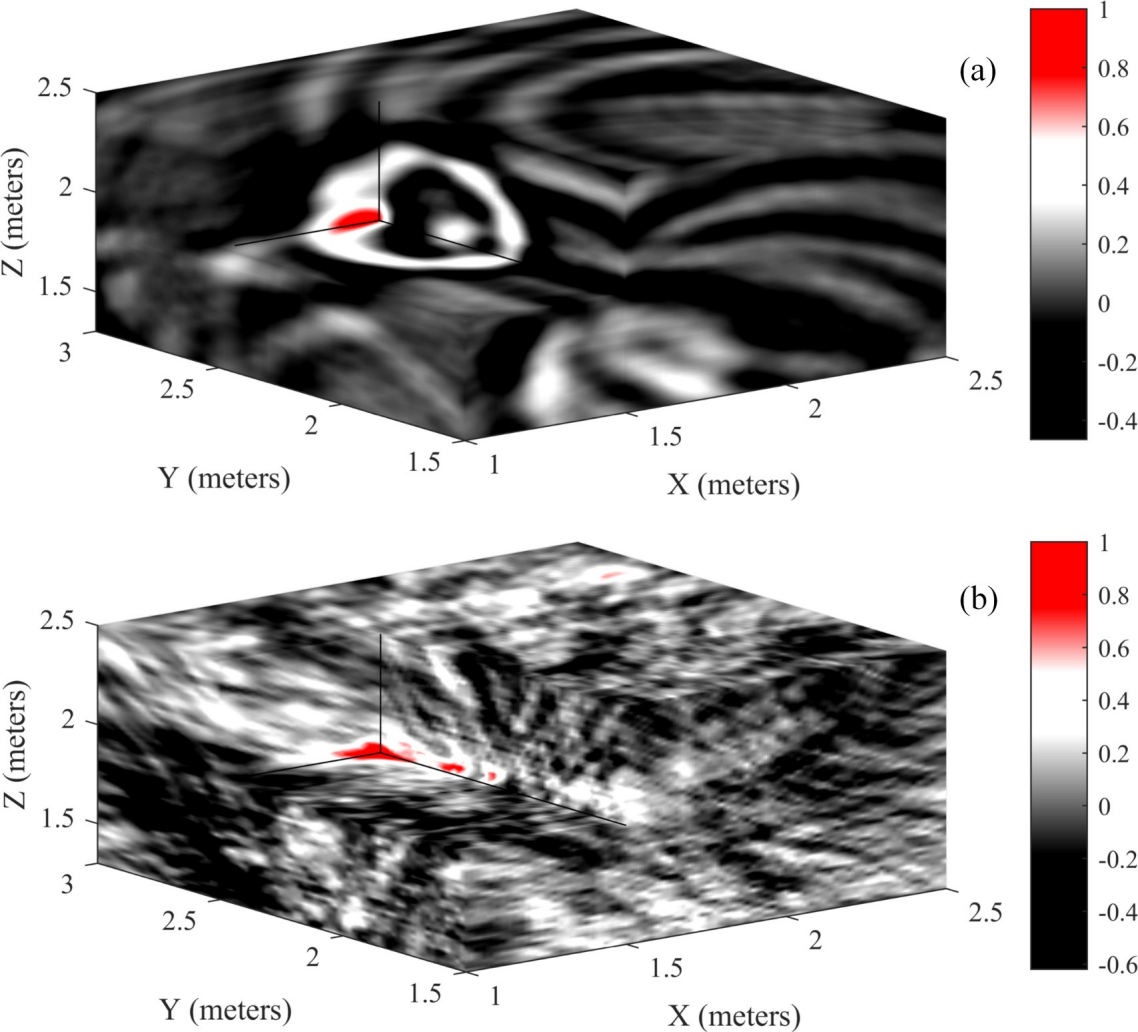
The spatial energy maps are presented using the SSA and SRP-PHAT. (a) the SSA and (b) the SRP-PHAT. It only shows the energy map of one of the two acoustic sources.

Similar to 2D positioning, the same room size, reflection coefficient, reverberation time, sampling frequency and spatial scanning resolution are used to simulate the room impulse response. The acoustic source is a wideband source, and Gaussian white noise is added to the composite signal to obtain noisy data with an SNR of 2 dB. The receivers are arranged at the eight corners and 50 cm from the corner. The four receivers on the floor are arranged 1 m from the floor, and the four receivers on the roof are arranged 0.3 cm from the roof. The positions of the two acoustic sources are p_s1_ = [2.0 m, 1.0 m, 1.7 m]^T^ and p_s2_ = [1.5 m, 2.5 m, 1.9 m]^T^. The spatial energy diagrams of the SSA and SRP-PHAT are shown in Fig 12, which shows that the energy aggregation of the SSA is relatively better than that of SRP-PHAT. The SSA only has a large energy distribution at the acoustic source position and has weaker energy at other positions. However, the SRP-PHAT has not only a large energy at the acoustic source position but also a close energy distribution at other positions, which results in difficulties in positioning. Fig 12 shows only the energy distribution of the p_s2_ source, which is similar to the energy distribution of p_s1_. However, the two methods are relatively more concentrated on the energy distribution of the p_s1_ location. The final 3D positioning results of the two methods are shown in Table 4, which shows that the positioning performance of the SSA at a high SNR is equivalent to that of the SRP-PHAT under the reverberation environment.

**Table 4:** 3D positioning results of the SSA and SRP-PHAT under the reverberation environment.

		Proposed method	SRP-PHAT
**p_s1_**	**(2.0, 1.0, 1.7)**	**(2.02, 1.0, 1.7)**	**(2.0, 1.02, 1.72)**
**p_s2_**	**(1.5, 2.5, 1.9)**	**(1.50, 2.52, 1.90)**	**(1.50, 2.46, 1.90)**

The above analysis demonstrates that the SSA can achieve high-precision positioning not only for a single source but also for multiple sources under the reverberation environment. Compared with the SRP-PHAT, especially for low SNR data, the SSA has more advantages and can obtain more accurate positioning results.

### Performance in real environments

**Fig 13:**
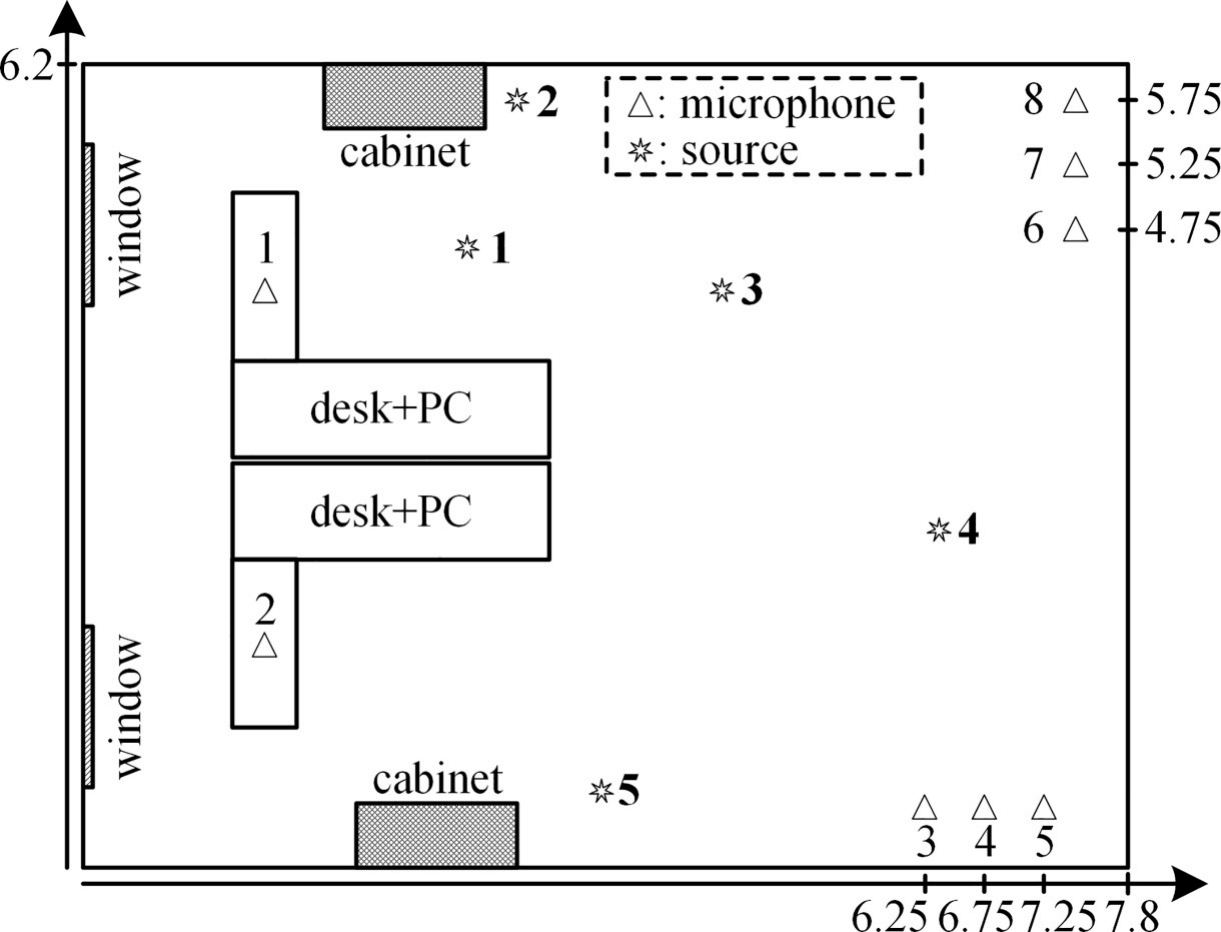
Room layout and experimental setup.

We used a regular office room of size 7.8 m × 6.2 m × 3.7 m with approximately T60 = 400 ms for 2D localization. Its ambient noise was low with an SNR of approximately 25 dB. Fig 13 shows the room layout with the experimental setup. The one-second speech was played back through the loudspeaker at each source point. The sampling frequency was 48 kHz.

**Fig 14:**
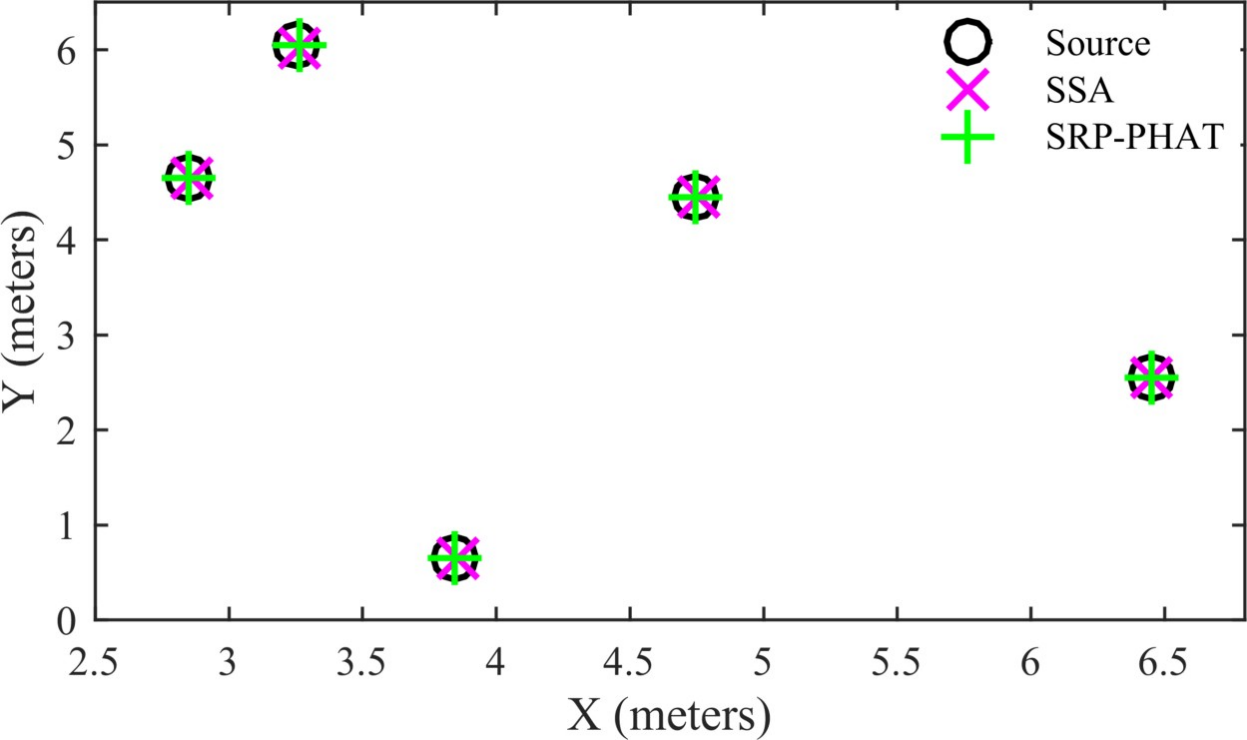
Comparison of the 2D position estimates for speech signals.

Five different positioning configurations of the sources were tested. All microphone pairs were used for localization in the SRP-PHAT. The search grid step was 1 cm for the SSA and SRP-PHAT. The estimated positions of the five sources by the SSA and SRP-PHAT are shown in Fig 14. It shows that the positioning accuracy of the SSA is almost the same as that of the SRP-PHAT for a speech signal with a high SNR. The localization errors of the SSA for the five sources are 0.01, 0.01, 0, 0, and 0. The localization errors of the SRP-PHAT for the five sources are 0.01, 0.02, 0.01, 0, and 0.01. The location accuracy of the SSA is slightly better than that of the SRP-PHAT.

## VI Conclusion

SRP-PHAT is one of the most effective and robust methods for source localization. However, the SRP-PHAT generates many local extrema in the SRP space. For the multi-source case, the SRP-PHAT cannot effectively locate an acoustic source that is far from the array. For high precision source localization, we propose a new algorithm based on the reciprocity theorem. The basic concept is to regard the sensor as a virtual source, which is equivalent to the back propagation of the wavefield to the spatial domain, and use it to form the energy map. The position of the energy peak corresponds to the source position. From a signal processing point of view, this approach can be understood as steered sampling. To improve the accuracy and stability of the SSA, the Gaussian function is introduced to weighted sampling. Moreover, the Gaussian function has an interpolation function to avoid interpolation calculations. Spatial energy maps with different SNRs can be obtained by adjusting the width parameters of the Gaussian function.

The SSA does not need to calculate the cross-correlation between microphone pairs and the number of adjustable sampling accumulations is less than that of the cross-correlation accumulations in the SRP-PHAT. Therefore, the computational complexity of the SSA is slightly lower than that of SRP-PHAT. However, these are not the primary factors that affect the computational complexity. The primary factor is the spatial grid search. In this respect, the computational complexity of the SSA is equivalent to that of the SRP-PHAT. Therefore, the SSA also has computational efficiency problems, similar to the SRP-PHAT. The same approaches used in the SRP-PHAT can be employed to reduce the computation cost, such as stochastic region contraction, coarse-to-fine region contraction and stochastic particle filtering. Because the SSA has only a few local extrema, the convergence rate of its optimization is faster than that of the SRP-PHAT. In the other aspects, when the SSA locates low-frequency signals, its spatial resolution is not as high as that of the SRP-PHAT. This is because the SRP-PHAT uses the PHAT transform. Ideally, a phase transform can sharpen GCC into the δ function. In that way, the peak of GCC is greatly sharpened. Therefore, the SRP-PHAT has a higher spatial resolution for low-frequency signal localization. Notably, the sharpening of the GCC peak also sharpens the noise and reduces the signal-to-noise ratio of the spatial energy map.

Similar to the SRP-PHAT, the SSA is also a one-step localization method that takes full advantage of all the information of the acquired signal. Compared with the SRP-PHAT, the SSA can achieve high-precision localization with a lower SNR. And the SSA is more suitable for narrow azimuth array signal localization than the SRP-PHAT. Furthermore, the SSA is more suitable than the SRP-PHAT for high-precision localization with fewer sensors.
